# Outcomes Associated With Use of a Kinin B2 Receptor Antagonist Among Patients With COVID-19

**DOI:** 10.1001/jamanetworkopen.2020.17708

**Published:** 2020-08-13

**Authors:** Frank L. van de Veerdonk, Ilse J. E. Kouijzer, Aline H. de Nooijer, Hans G. van der Hoeven, Coen Maas, Mihai G. Netea, Roger J. M. Brüggemann

**Affiliations:** 1 Radboudumc Institute for Molecular Life Sciences, Department of Internal Medicine, Radboudumc, Nijmegen, the Netherlands; 2 Radboudumc Center for Infectious Diseases, Nijmegen, the Netherlands; 3 Department of Intensive Care Medicine, Radboudumc, Nijmegen, the Netherlands; 4 Department of Intensive Care Medicine, University Medical Center Utrecht, Utrecht, the Netherlands; 5 Life and Medical Sciences Institute, Department for Genomics & Immunoregulation, University of Bonn, Bonn, Germany; 6 Radboud Institute for Health Sciences, Department of Pharmacy, Radboudumc, Nijmegen, the Netherlands

## Abstract

This case-control study examines the association between receipt of the bradykinin 2 (B2) receptor antagonist icatibant and improved oxygenation in patients with coronavirus disease 2019 (COVID-19).

## Introduction

Pulmonary edema is a prominent feature in patients with severe coronavirus disease 2019 (COVID-19). Severe acute respiratory syndrome coronavirus 2 (SARS-CoV-2) enters the cell via angiotensin-converting enzyme 2 (ACE2).^1^ ACE2 is involved in degrading the kinin des-Arg^9^-bradykinin, a potent vasoactive peptide that can cause vascular leakage. Loss of ACE2 might lead to plasma leakage and further activation of the plasma kallikrein-kinin system with more bradykinin formation that could contribute to pulmonary angioedema via stimulation of bradykinin 2 receptors.^2^ We investigated whether treatment with the bradykinin 2 receptor antagonist icatibant in patients with COVID-19 could be used as a treatment strategy.

## Methods

This case-control study was approved by CMO region Arnhem-Nijmegen, the local ethical committee, which granted a waiver of consent because treatment concerned a licensed drug that would be given in an off-label setting. Informed consent was obtained in all patients. This study followed the Strengthening the Reporting of Observational Studies in Epidemiology (STROBE) reporting guideline. Patients with COVID-19 were admitted from March to May 2020. We included 10 patients for treatment with 3 doses of 30 mg of icatibant (Firazyr; Shire Pharmaceuticals Ireland Limited) by subcutaneous injection at 6-hour intervals.^3^ Patients were eligible for icatibant treatment if they had confirmed SARS-CoV-2 by polymerase chain reaction assay, an oxygen saturation of less than 90% without supplemental oxygen, needed 3 L/min supplemental oxygen or more, and had a computed tomography severity score of 7 or greater.^4^ Patients with acute ischemic events at time of eligibility were excluded. For 9 patients who received icatibant on the ward, 2 matched control patients admitted prior to approval of this treatment were selected. Control patients with COVID-19 were matched on the factors sex, age, body mass index, and day of illness. One patient started receiving icatibant in the intensive care unit and was transferred to the ward with high-flow oxygen supplementation within 24 hours and discharged on day 7. We did not identify a matched control for this patient, so we were not able to evaluate the association of icatibant with outcomes for this individual. A change in oxygen need and oxygenation expressed as absolute number of liters per hour served as the primary outcome variable. Secondary outcomes included changes in D-dimer (dimerized plasmin fragment D), fever, and safety.

## Results

Nine cases were matched to 18 controls. The mean (SD) age was 55 (12.8) years for cases and 58 (10.5) years for controls. Most cases (9 of 10 [90%]) and controls (16 of 18 [90%]) were men. Patient and matched control characteristics are shown in the Table. Nine patients were prescribed icatibant on the ward. In all 9 patients, there was a marked decrease in oxygen supplementation (Figure). After 3 injections of icatibant, 4 patients (44%) were no longer oxygen dependent within 10 to 35 hours. In 5 patients (56%), there was a substantial decrease of oxygen supplementation after treatment with icatibant (Figure). Overall, in 8 of 9 patients (89%) treated with icatibant, a reduction of 3 L/min in oxygen supplementation or greater after 24 hours was observed (Figure). Of 18 matched controls, only 3 (17%) showed a spontaneous reduction in oxygen supplementation of 3 L/min or greater within 24 hours. We noted that in 3 patients treated with icatibant there was a resurgence in the need for oxygen supplementation. Icatibant treatment was well tolerated in all 10 patients who received the drug. There were no severe adverse events. There was no clear association with D-dimer concentrations and fever.

**Table.** Characteristics of 10 COVID-19 Patients With Icatibant Treatment and Matched Controls

Participant No./sex/age (decade)	BMI	Days of illness^a^	D-dimer, ng/mL		Temperature at admission, °C
			At admission	24 h^b^	
**Cases**					
1/M/30s	23	12	3760	11 530	39.9
2/M/50s	30	15	650	<500	40.0
3/M/60s	29	10	NA	690	38.4
4/M/40s	27	12	NA	920	40.0
5/M/60s	26	11	530	630	39.3
6/M/70s	24	7	NA	<500	39.1
7/M/30s	25	8	1730	3560	38.3
8/M/60s	33	10	870	1240	37.1
9/F/50s	32	11	1590	1030	40.1
10/M/40s	28	15	1600	1000	37.1
**Controls**					
1a/M/30s	23	2	1360	NA	38.1
1b/M/40s	27	13	1750	NA	37.8
2a/M/50s	26	15	1360	780	36.9
2b/M/50s	27	15	1500	NA	39.9
3a/M/60s	25	9	NA	NA	37.1
3b/M/70s	25	9	3710	3710	39.2
4a/M/50s	28	10	760	700	39.4
4b/M/50s	27	8	620	500	39.6
5a/M/60s	22	11	1520	940	37.1
5b/M/60s	27	11	1550	1340	39.4
6a/M/60s	25	6	700	840	38.2
6b/M/70s	29	8	3660	2080	37.7
7a/M/40s	21	5	33 790	7060	36.9
7b/M/40s	31	12	2210	1660	38.0
8a/F/60s	36	4	1740	NA	38.7
8b/F/60s	27	11	510	600	38.0
9a/M/50s	22	10	<500	<500	39.5
9b/M/50s	22	14	3300	2150	38.8

Abbreviations: BMI, body mass index (calculated as weight in kilograms divided by height in meters squared); COVID-19, coronavirus disease 2019; D-dimer, dimerized plasmin fragment D; F, female; M, male; NA, not applicable.

SI conversion: To convert D-dimer to nmol/L, multiply by 5.476.

^a^ Days of illness at start icatibant or 24 hours after admission (controls).

^b^ D-dimer 24 hours after icatibant or 48 hours after admission (controls).

**Figure.**
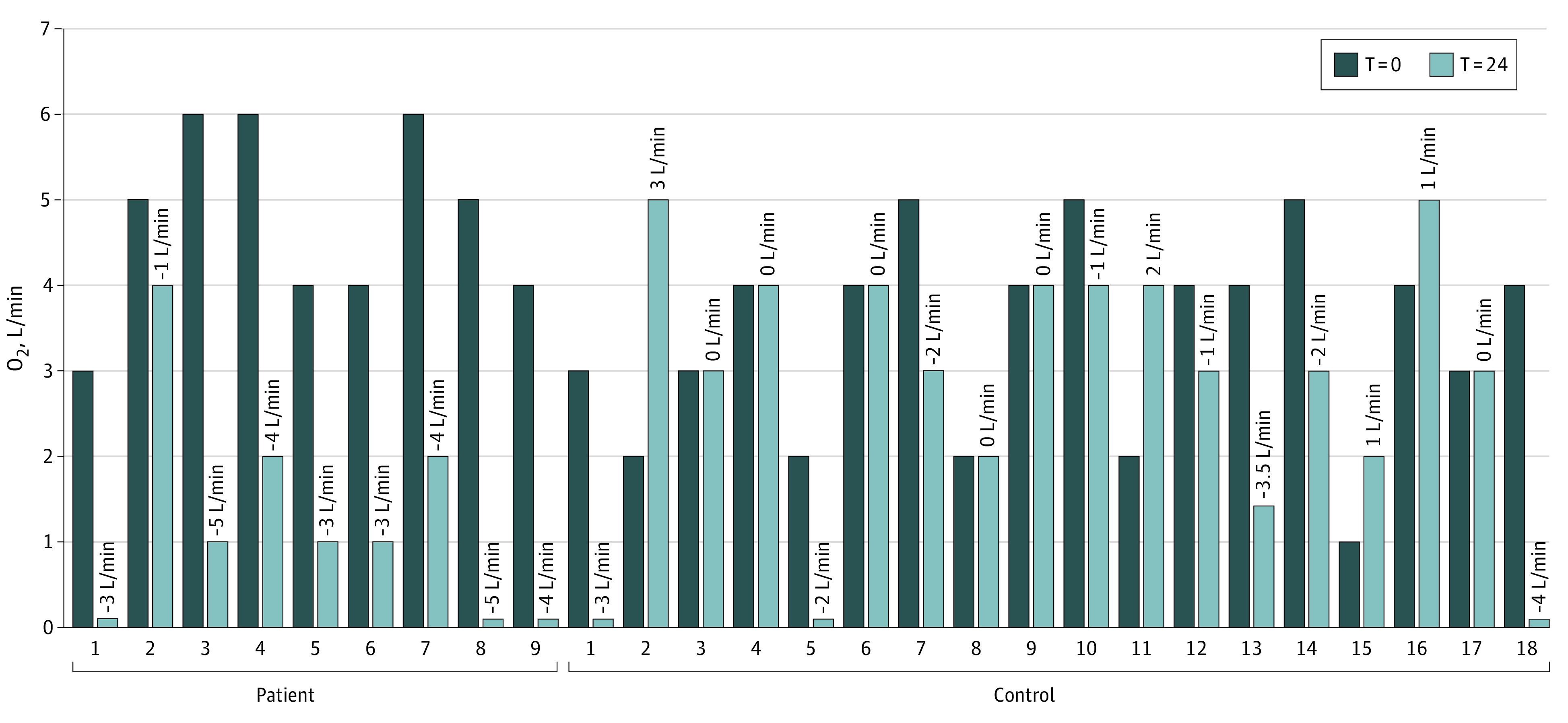
Changes in Oxygen Supplementation at Baseline and 24 Hours in Individual Patients and Controls Oxygen supplementation in individual patients (n = 9) and matched controls (n = 18).

## Discussion

This study found evidence of an association between receipt of icatibant and improved oxygenation, suggesting that targeting the kallikrein-kinin system in patients with COVID-19, especially in the early stages of disease when patients are hypoxic and are admitted to the hospital, might be beneficial. An important limitation of the current study is that it is exploratory and not a randomized clinical trial. The observed resurgence of oxygen need in some patients after icatibant may be due to icatibant’s short half life of about 2 hours.^3^ We propose that treatment strategies targeting the kallikrein-kinin system should be investigated in randomized trials for patients with COVID-19.
